# School Start Times for Middle School and High School Students — United States, 2011–12 School Year

**DOI:** 10.15585/mmwr.mm6430a1

**Published:** 2015-08-07

**Authors:** Anne G. Wheaton, Gabrielle A. Ferro, Janet B. Croft

**Affiliations:** 1 Division of Population Health, National Center for Chronic Disease Prevention and Health Promotion, CDC

Adolescents who do not get enough sleep are more likely to be overweight (1); not engage in daily physical activity (2); suffer from depressive symptoms (2); engage in unhealthy risk behaviors such as drinking, smoking tobacco, and using illicit drugs (2); and perform poorly in school (3). However, insufficient sleep is common among high school students, with less than one third of U.S. high school students sleeping at least 8 hours on school nights (4). In a policy statement published in 2014, the American Academy of Pediatrics (AAP) urged middle and high schools to modify start times as a means to enable students to get adequate sleep and improve their health, safety, academic performance, and quality of life (5). AAP recommended that “middle and high schools should aim for a starting time of no earlier than 8:30 a.m.” (5). To assess state-specific distributions of public middle and high school start times and establish a pre-recommendation baseline, CDC and the U.S. Department of Education analyzed data from the 2011–12 Schools and Staffing Survey (SASS). Among an estimated 39,700 public middle, high, and combined schools* in the United States, the average start time was 8:03 a.m. Overall, only 17.7% of these public schools started school at 8:30 a.m. or later. The percentage of schools with 8:30 a.m. or later start times varied greatly by state, ranging from 0% in Hawaii, Mississippi, and Wyoming to more than three quarters of schools in Alaska (76.8%) and North Dakota (78.5%). A school system start time policy of 8:30 a.m. or later provides teenage students the opportunity to achieve the 8.5–9.5 hours of sleep recommended by AAP (5) and the 8–10 hours recommended by the National Sleep Foundation (6).

Every few years, the U.S. Department of Education conducts SASS, which provides data on the condition of elementary and secondary education in the United States. SASS consists of several questionnaires, including those tailored to schools, teachers, principals, school districts, and library media centers. SASS is a mail-based survey, with telephone and field follow-up, and uses a stratified probability sample design.† For the 2011–12 school year, the sample included about 10,250 traditional public schools and 750 public charter schools, with a unit response rate for public schools of 72.5%. As part of the school questionnaire in the 2011–12 school year, respondents were asked, “At what time do most of the students in this school begin the school day?” Because AAP recommends school start times of 8:30 a.m. or later for both middle schools and high schools, the analyses in this report include public middle schools, high schools, and schools with combined grades. Average start time (with standard error) and percentage distribution of start times were calculated by school level and state. Results are weighted to reflect the complex sample design and to account for nonresponse and other adjustments.

Among an estimated 39,700 U.S. public middle, high, and combined schools (with an estimated total enrollment of 26.3 million students), the average start time was 8:03 a.m. Forty-two states reported that 75%–100% of their public schools had early start times (before 8:30 a.m.) (Figure). Overall, only 17.7% of public schools (with an estimated total enrollment of 4.2 million students), started school at 8:30 a.m. or later (Table). The proportion was lowest for high schools (14.4%) and highest for combined schools (23.4%). The percentage of schools that started at 8:30 a.m. or later varied greatly by state, ranging from 0% in Hawaii, Mississippi, and Wyoming to 76.8% in Alaska and 78.5% in North Dakota. North Dakota and Alaska also reported the latest average school start times (8:31 a.m. and 8:33 a.m., respectively), whereas Louisiana reported the earliest average school start time (7:40 a.m.) and the largest percentage of schools starting before 7:30 a.m. (29.9%).

## Discussion

Obtaining adequate sleep is important for achieving optimal health. Among adolescents, insufficient sleep has been associated with adverse risk behaviors (2), poor health outcomes (1), and poor academic performance (3). In view of these negative outcomes, the high prevalence of insufficient sleep among high school students is of substantial public health concern. *Healthy People 2020* includes a sleep objective for adolescents: to “increase the proportion of students in grades 9 through 12 who get sufficient sleep (defined as 8 or more hours of sleep on an average school night).”§ However, the proportion of students who get enough sleep has remained approximately 31% since 2007 (4), the first year that the national Youth Risk Behavior Survey included a question about sleep, meaning that more than two thirds of high school students do not get enough sleep. Multiple contributors to insufficient sleep in this population might exist. In puberty, biological rhythms commonly shift so that adolescents become sleepy later at night and need to sleep later in the morning (7). These biological changes are often combined with poor sleep hygiene (including irregular bedtimes and the presence of televisions, computers, or mobile phones in the bedroom) (8). During the school week, the chief determinant of wake times is school start time (9). The combination of delayed bedtimes and early school start times results in inadequate sleep for a large portion of the adolescent population.

Citing evidence of the benefits of delayed school start times for adolescents, AAP released a policy statement in 2014 that encouraged middle and high schools to modify start times to enable students to get sufficient sleep and subsequently improve their health, safety, academic performance, and quality of life (5). AAP recommended that schools start at 8:30 a.m. or later (5), but this was the case in only one in six U.S. public middle and high schools, with substantial variation by state. Because school start times are determined at the district or even individual school level, local stakeholders have the most influence on whether start times change in their communities.

Groups seeking to delay school start times in their district often face resistance. Common barriers to delaying school start times include concerns about increased transportation costs because of changes in bus schedules; potential for traffic congestion for students and faculty; difficulty in scheduling after-school activities, especially athletic programs; and lack of education in some communities about the importance of sleep and school start times.¶ Advocates for delayed start times might benefit from 1) becoming familiar with research about the negative impact of insufficient sleep and early start times on adolescents’ health, well-being, and academic performance; 2) identification of persons who might be impacted by the decision to delay start times, including parties involved in transportation and school athletic programs, as well as students, teachers, and school staff; and 3) preparing responses to common arguments against delaying start times. Many school systems have successfully overcome barriers to delay start times.**

Among the possible public health interventions for increasing sufficient sleep among adolescents, delaying school start times has the potential for the greatest population impact by changing the environmental context for students in entire school districts. However, a late school start time does not preclude the need for other interventions that have the potential to improve the sleep of adolescents. Health care providers who treat adolescents, both in and outside of school settings, should educate patients and parents about the importance of adequate sleep, as well as factors that contribute to insufficient sleep among adolescents. Parents can help their children practice good sleep hygiene (i.e., habits that help promote good sleep). A regular bedtime and rise time, including on weekends, is recommended for everyone, whether they are children, adolescents, or adults.†† In addition, adolescents with parent-set bedtimes usually get more sleep than those whose parents do not set bedtimes (8). Adolescents who are exposed to more light (such as room lighting or from electronics) in the evenings are less likely to get enough sleep (8). Technology use (e.g., computers, video gaming, or mobile phones) might also contribute to late bedtimes (8) and parents might consider implementing a “media curfew” or removing these technologies from the bedroom. Finally, parents might benefit themselves and their children by setting a good example. Adolescent sleep habits tend to reflect their parents’ sleep habits (10).

**Summary**

What is already known on this topic?

The American Academy of Pediatrics (AAP) has urged middle and high schools to modify school start times to enable adolescent students to get sufficient sleep and improve their health, safety, academic performance, and quality of life. AAP recommends that schools aim to start no earlier than 8:30 a.m.

What is added by this report?

During the 2011–12 school year, before publication of the new AAP recommendations, only 17.7% of public middle and high schools in the United States started school at 8:30 a.m. or later. The percentage varied greatly by state, ranging from 0% in Hawaii, Mississippi, and Wyoming to more than three quarters of schools in Alaska (76.8%) and North Dakota (78.5%).

What are the implications for public health practice?

School start time policies are established at the district and individual school levels. Educating parents and school system decision-makers about the impact of sleep deprivation on adolescent health and academic performance might lead to adoption of later start times.

## Figures and Tables

**FIGURE:**
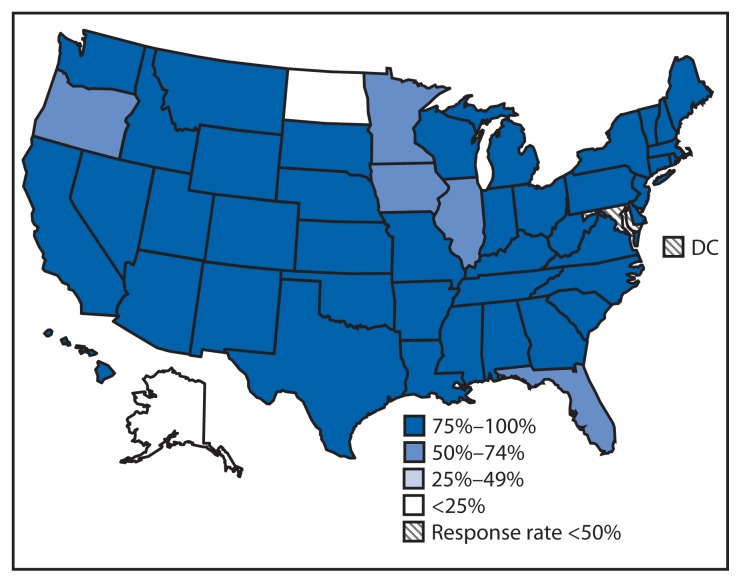
Percentage of public schools* with early school start times (before 8:30 a.m.), by state — Schools and Staffing Survey, United States, 2011–12 school year **Source:** U.S. Department of Education, National Center for Education Statistics, Schools and Staffing Survey, public school data file, 2011–12. Additional information available at http://nces.ed.gov/surveys/sass/overview.asp. * Includes middle, high, and combined schools. Middle schools include any schools with no grade lower than 5 and no grade higher than 8. High schools include any school with no grade lower than 7 and at least one grade higher than 8. Combined schools include any schools with at least one grade lower than 7 and at least one grade higher than 8, or with all students in ungraded classrooms.

**TABLE:** Average start time and percentage distribution of start times for public middle, high, and combined schools,* by school level and state — Schools and Staffing Survey 2011–12 school year

School level and state	Estimated no. of public middle, high, and combined schools		Estimated no. of students in public middle, high, and combined schools		Average start time (a.m.)¶		Percentage distribution† of public middle, high, and combined school start times							
	
					Before 7:30 a.m.		7:30 a.m. to 7:59 a.m.		8:00 a.m. to 8:29 a.m.		8:30 a.m. or later		8:30 a.m. or later	
						
	No.	(SE)	No.	(SE)	Time	(SE)§	%	(SE)	%	(SE)	%	(SE)	%	(SE)
**Total**	**39,700**	**(390)**	**26,284,000**	**(613,100)**	**8:03**	**(1)**	**6.7**	**(0.4)**	**31.9**	**(0.8)**	**43.7**	**(0.8)**	**17.7**	**(0.7)**
**School level**														
Middle	13,990	(169)	8,674,000	(135,800)	8:04	(1)	4.8	(0.7)	35.9	(1.3)	40.4	(1.1)	18.9	(1.0)
High	18,360	(434)	14,995,000	(413,600)	7:59	(1)	9.5	(0.6)	33.0	(1.1)	43.1	(1.1)	14.4	(0.9)
Combined	7,350	(571)	2,615,000	(300,600)	8:08	(3)	3.5	(0.7)	21.6	(2.2)	51.5	(2.6)	23.4	(2.7)
**State**														
Alabama	680	(39)	344,000	(31,100)	7:49	(2)	6.4	(2.2)††	57.8	(4.4)	34.0	(5.3)	—**	—
Alaska	—**	—	—**	—	8:33	(8)	0.0	—§§	11.6	(3.8)††	11.6	(4.8)††	76.8	(7.8)
Arizona	860	(159)	506,000	(53,100)	8:03	(3)	8.1	(2.9)††	23.3	(6.6)	47.3	(5.8)	21.3	(5.0)
Arkansas	450	(28)	292,000	(30,300)	8:01	(1)	—**	—	29	(4.7)	63.0	(4.7)	7.3	(2.0)
California	3,880	(219)	3,303,000	(146,300)	8:07	(2)	3.5	(0.9)	27.7	(3.1)	47.6	(3.3)	21.2	(2.9)
Colorado	730	(84)	527,000	(51,700)	7:54	(2)	16.9	(5.1)	31.3	(6.6)	40.9	(5.1)	10.9	(2.6)
Connecticut	380	(24)	260,000	(23,900)	7:46	(2)	13.8	(2.9)	57.4	(4.2)	24.0	(3.8)	4.8	(2.1)††
Delaware	090	(4)	63,000	(4,900)	7:42	(3)	24.0	(5.3)	51.9	(6.3)	16.6	(4.6)	7.5	(3.0)††
District of Columbia	—**	—	—**	—	—**	—	—**	—	—**	—	—**	—	—**	—
Florida	1,570	(100)	1,406,000	(111,400)	8:17	(3)	19.5	(2.5)	18.6	(2.4)	19.3	(2.9)	42.6	(3.8)
Georgia	1,030	(24)	955,000	(77,500)	8:09	(2)	—**	—	28.7	(4.3)	43.9	(4.6)	24.0	(3.4)
Hawaii	—**	—	—**	—	8:03	(3)	0.0	—§§	42.5	(17.3)††	57.5	(17.3)††	0.0	—§§
Idaho	370	(182)	157,000	(40,300)	8:13	(28)	0.0	—§§	20.9	(7.5)††	58.3	(14.5)	—**	—
Illinois	1,590	(48)	1,008,000	(145,200)	8:13	(3)	—**	—	19.7	(3.4)	48.7	(5.5)	28.4	(6.0)
Indiana	740	(27)	559,000	(43,800)	7:58	(2)	—**	—	41.8	(3.2)	45.1	(4.0)	10.2	(2.7)
Iowa	550	(35)	249,000	(31,300)	8:23	(6)	0.0	—§§	6.3	(2.0)††	66.3	(7.2)	27.4	(7.6)
Kansas	540	(20)	204,000	(20,000)	8:00	(1)	—**	—	26.5	(3.5)	71.5	(3.7)	—**	—
Kentucky	710	(32)	358,000	(33,100)	8:03	(4)	8.6	(4.2)††	24.8	(4.0)	49.0	(5.8)	17.5	(4.0)
Louisiana	630	(32)	316,000	(33,100)	7:40	(2)	29.9	(4.8)	53.1	(4.9)	12.1	(3.5)	—**	—
Maine	240	(5)	105,000	(5,500)	7:53	(3)	6.6	(1.9)	53.1	(5.1)	32.8	(4.8)	7.5	(3.6)††
Maryland	—**	—	—**	—	—**	—	—**	—	—**	—	—**	—	—**	—
Massachusetts	700	(58)	527,000	(48,600)	7:53	(4)	8.0	(3.6)††	53.3	(6.1)	27.2	(5.1)	11.5	(5.4)††
Michigan	1,540	(47)	891,000	(59,100)	7:54	(2)	9.5	(2.1)	43.6	(3.6)	39.0	(3.5)	7.9	(2.2)
Minnesota	1,100	(58)	522,000	(43,100)	8:18	(3)	0.9	(0.4)††	18.8	(2.6)	46.7	(3.7)	33.6	(3.5)
Mississippi	570	(23)	272,000	(18,600)	7:47	(2)	12.4	(3.7)††	58.3	(4.3)	29.3	(4.3)	0.0	—§§
Missouri	900	(37)	530,000	(28,700)	7:54	(1)	6.7	(1.7)	39.0	(3.9)	51.0	(3.9)	3.2	(1.4)††
Montana	220	(15)	78,000	(8,200)	8:13	(2)	0.0	—§§	5.8	(2.1)††	80.9	(6.1)	13.4	(5.5)††
Nebraska	370	(26)	150,000	(19,200)	8:07	(1)	0.0	—§§	8.0	(2.5)††	88.9	(2.4)	3.0	(1.4)††
Nevada	260	(12)	276,000	(20,900)	7:51	(3)	18.0	(3.0)	30.7	(5.5)	38.2	(6.0)	13.1	(3.6)
New Hampshire	180	(18)	116,000	(7,800)	7:46	(2)	11.6	(3.2)	64.4	(5.7)	19.7	(4.4)	—**	—
New Jersey	870	(52)	698,000	(45,200)	8:00	(2)	6.7	(2.0)	37.2	(4.5)	41.2	(4.7)	14.9	(3.6)
New Mexico	310	(99)	151,000	(47,000)	8:10	(3)	1.6	(0.7)††	24.1	(5.8)	53.9	(10.2)	20.4	(5.9)
New York	2,070	(108)	1,670,000	(149,100)	7:59	(2)	7.7	(3.1)††	31.6	(2.9)	49.6	(3.4)	11.0	(2.5)
North Carolina	1,120	(35)	768,000	(88,900)	8:03	(2)	—**	—	36.6	(5.0)	45.3	(5.4)	15.2	(4.2)
North Dakota	220	(9)	67,000	(5,000)	8:31	(1)	0.0	—§§	2.8	(1.2)††	18.7	(3.2)	78.5	(3.4)
Ohio	1,640	(73)	1,061,000	(60,800)	7:52	(2)	13.1	(2.0)	45.3	(4.3)	29.3	(3.7)	12.3	(3.0)
Oklahoma	700	(27)	356,000	(29,000)	8:10	(2)	0.0	—§§	12.0	(2.8)	77.6	(3.9)	10.4	(2.8)
Oregon	480	(25)	282,000	(21,100)	8:14	(3)	—**	—	25.2	(3.8)	45.0	(4.1)	28.9	(4.2)
Pennsylvania	1,280	(145)	1,001,000	(189,700)	7:48	(2)	13.0	(3.0)	51.3	(6.6)	32.6	(7.9)	3.1	(1.3)††
Rhode Island	100	(10)	68,000	(6,200)	7:50	(4)	24.8	(6.1)	27.5	(7.9)	40.3	(9.2)	—**	—
South Carolina	500	(9)	411,000	(26,400)	8:03	(2)	—**	—	35.3	(6.5)	50.9	(6.8)	12.3	(3.7)
South Dakota	230	(11)	78,000	(5,200)	8:13	(2)	—**	—	6.6	(2.7)††	77.7	(4.2)	14.8	(4.9)††
Tennessee	760	(47)	533,000	(31,000)	7:57	(3)	13.3	(3.4)	29.4	(4.7)	40.0	(5.1)	17.2	(3.5)
Texas	3,940	(183)	2,556,000	(254,700)	8:05	(2)	3.1	(1.2)††	28.3	(3.4)	46.3	(3.5)	22.4	(2.7)
Utah	410	(22)	297,000	(45,200)	8:05	(3)	0.0	—§§	33.1	(5.3)	49.6	(5.9)	17.3	(5.9)††
Vermont	100	(2)	46,000	(2,600)	8:05	(2)	—**	—	34.1	(5.1)	48.0	(4.8)	15.1	(3.0)
Virginia	850	(17)	555,000	(37,700)	8:04	(2)	10.0	(2.6)	26.6	(4.4)	42.6	(4.4)	20.8	(3.6)
Washington	930	(35)	526,000	(42,300)	8:08	(2)	6.4	(1.9)††	24.2	(3.8)	50.2	(4.6)	19.3	(3.5)
West Virginia	300	(5)	160,000	(7,000)	7:54	(2)	11.1	(2.0)	33.9	(3.3)	47.9	(4.0)	7.1	(2.3)††
Wisconsin	860	(37)	423,000	(44,200)	7:59	(3)	2.3	(1.0)††	48.2	(5.4)	39.1	(4.3)	10.4	(4.4)††
Wyoming	130	(8)	50,000	(4,300)	7:59	(1)	0.0	—§§	41.1	(5.2)	58.9	(5.2)	0.0	—§§

**Source:** U.S. Department of Education, National Center for Education Statistics, Schools and Staffing Survey (SASS), “Public School Data File,” 2011–12.

**Abbreviation:** SE = standard error.

* Middle schools include any schools with no grade lower than 5 and no grade higher than 8. High schools include any school with no grade lower than 7 and at least one grade higher than 8. Combined schools include any schools with at least one grade lower than 7 and at least one grade higher than 8, or with all students in ungraded classrooms.

† Detail may not sum to totals because of rounding and because some data are not shown.

§ SE of average start time is expressed in minutes.

¶ Schools with afternoon start times were not included in analysis.

** Reporting standards not met. Relative standard error ≥0.5 or the response rate <50%.

†† Interpret data with caution. 0.3 ≤ relative standard error < 0.5.

§§ Rounds to zero. SE is not applicable.

## Footnotes

* Middle schools include any schools with no grade lower than 5 and no grade higher than 8. High schools include any school with no grade lower than 7 and at least one grade higher than 8. Combined schools include any schools with at least one grade lower than 7 and at least one grade higher than 8, or with all students in ungraded classrooms.

† Additional information available at http://nces.ed.gov/surveys/sass/overview.asp and http://nces.ed.gov/statprog/handbook/sass_surveydesign.asp. Questions about SASS can be directed to Chelsea Owens at chelsea.owens@ed.gov.

§ Information on *Healthy People 2020* sleep objectives is available at http://www.healthypeople.gov/2020/topics-objectives/topic/sleep-health.

¶ A discussion of common barriers faced by proponents of delayed school start times is available at http://sleepfoundation.org/sleep-news/eight-major-obstacles-delaying-school-start-times.

** Several case studies that describe how this was done were compiled by the National Sleep Foundation and are available at http://www.startschoollater.net/case-studies.html.

†† Information on healthy sleep habits, often referred to as good “sleep hygiene”, is available at http://sleepfoundation.org/sleep-tools-tips/healthy-sleep-tips.
